# Effects of Elevated Temperature on Root System Development of Two Lupine Species

**DOI:** 10.3390/plants11020192

**Published:** 2022-01-12

**Authors:** Virgilija Gavelienė, Sigita Jurkonienė, Elžbieta Jankovska-Bortkevič, Danguolė Švegždienė

**Affiliations:** Nature Research Centre, Institute of Botany, Akademijos Str. 2, 08412 Vilnius, Lithuania; elzbieta.jankovska@gamtc.lt (E.J.-B.); dangasv@gmail.com (D.Š.)

**Keywords:** gravitropic angle of curvature, initial root, invasiveness, lateral root number, primary root, root system architecture, simulated warming

## Abstract

The aim of this study was to assess the effect of elevated temperature on the growth, morphology and spatial orientation of lupine roots at the initial stages of development and on the formation of lupine root architecture at later stages. Two lupine species were studied—the invasive *Lupinus polyphyllus* Lindl. and the non-invasive *L. luteus* L. The plants were grown in climate chambers under 25 °C and simulated warming at 30 °C conditions. The angle of root curvature towards the vector of gravity was measured at the 48th hour of growth, and during a 4-h period after 90° reorientation. Root biometrical, histological measurements were carried out on 7-day-old and 30-day-old plants. The elevation of 5 °C affected root formation of the two lupine species differently. The initial roots of *L. polyphyllus* were characterized by worse spatial orientation, reduced growth and reduced mitotic index of root apical meristem at 30 °C compared with 25 °C. The length of primary roots of 30-day-old lupines and the number of lateral roots decreased by 14% and 16%, respectively. More intense root development and formation were observed in non-invasive *L. luteus* at 30 °C. Our results provide important information on the effect of elevated temperature on the formation of root architecture in two lupine species and suggest that global warming may impact the invasiveness of these species.

## 1. Introduction

The world is experiencing ongoing global climate change, which can have serious consequences on plants, including changes in the availability of certain nutrients. For understanding the effects of climate warming on plant root systems, particularly their spatial distribution, it is essential to predict plant performance and community recovery in a warming climate. Compared with shoots, much less is known about how roots, especially root system architecture (RSA), may respond to elevated temperature. In addition, limited information is available on the specificities of the effects of elevated temperatures on the development of the root system in invasive plants. How does an increase in temperature change the intensity and the direction of root formation? To answer this question, researchers have compared the responses of plants with different RSAs in their studies [1,2]. The ability of a plant to take up nutrients is closely associated with the size and morphology of its root system [1,3]. Any changes in the growth or morphological modifications of root systems may provoke undesirable consequences in nutrient uptake [4]. It is recognized that many aspects of plant metabolism are accelerated by elevated temperatures [5,6]. Other environmental factors such as water, nutrients and temperature also have a strong influence on root structure [7]. Roots need an optimal temperature range to have a proper growth rate and function. In general, the optimal root temperature tends to be lower than the optimal shoot temperature [8,9]. It is evident that the optimum root temperature of plants varies depending on the species. Within this range, higher temperatures are generally associated with modified root-to-shoot ratios, while further increases in temperature would reduce root development and cause a change in RSA, thus reducing the root-to-shoot ratio [10]. For instance, some plants tend to produce more extensive root systems in elevated temperatures. An increase in temperature slows down lateral root growth in adult maize plants and promotes the development of long axial roots to reach deeper soil layers for water [11,12]. However, in potatoes, the initiation and elongation of adventitious and lateral roots were inhibited by increasing temperature. Another effect of warmer soil on potatoes is the swelling of the root cap meristem and the bending of the root tip. The alteration of root growth in these plants appeared due to a reduced rate of cell division [13,14]. Similarly, in sorghum, the high root zone temperature reduced the rate of root elongation and cell production rate [15]. The response of RSA to elevated temperature can be species-specific, as different species often have different optimum temperatures for root growth [16,17]. Literature data show that the effect of increasing temperature on root growth of plant seedlings can be promotive, inhibitive or first promotive then inhibitive after an optimum temperature is reached [18,19]. Even for species sharing the same habitat, their RSA can have species-specific responses to increased temperature [20]. Differences in the RSA of plant species may determine the intensity and direction of root formation in response to elevated temperatures. At high temperatures, the negative root response may have been intensified, with a competitive advantage going to species with larger and more rapidly forming roots.

Literature data indicate that greater root resilience plays a key role in plants adapting to high temperatures [21,22,23] in all stages of root development, including tropisms and the formation of new organs [24,25]. Furthermore, the oriented plant growth, which is collectively referred to as tropism [26,27], is influenced by various environmental factors, such as light, temperature, water and gravity. Gravitropism is an important tropic response that triggers asymmetric cell elongation in plant organs in response to gravity. It proceeds through three sequential steps: gravity perception, signal transduction and asymmetric cell elongation in the responding plant organs [28,29]. The roots grow downward, and the shoots grow upward, showing positive and negative gravitropic responses, respectively [30,31]. The well-known Cholodny-Went hypothesis illustrates that gravitropic stimuli result in differential cell elongation in the responding organs [32,33,34,35]. It has been shown that gravitropic perception occurs in the columella cells in the roots upon gravity stimulation [36,37]. The gravitropic response of plant organs is influenced by a variety of environmental signals. The best understood are the effects of light and temperature. Many scientists agree that climate change will alter habitat biodiversity and increase vulnerability to invasion. However, there is little information on the impact of potentially increasing global temperatures on the growth and development of alien plant species at the early stages of development. Moreover, one of the selected lupine species is invasive in Lithuania, *L. polyphyllus*, and there is very limited information on the specificities of the effect of elevated temperatures on the root system development of invasive plants. Therefore, in this research, RSA traits of seedlings of two lupine species (*L. polyphyllus* and *L. luteus*) with different spreading performances for understanding their responses to temperature change were studied. We hypothesized that increased temperature may differentially affect root growth, spatial orientation and root architecture of non-native plant species, thereby influencing them to become invasive. Studies on plant root system adaptive responses to altered temperature can provide the knowledge needed for the efficient management of invasive species. Thus, the goal of the current study was to investigate root growth, morphology and spatial orientation of two alien lupine species during the early growth stage at the elevated temperature.

## 2. Results

### 2.1. The Initial Root Growth at 25 °C and 30 °C

#### 2.1.1. Angle of Curvature of Initial Roots at 25 °C and 30 °C

**Figure 1:**
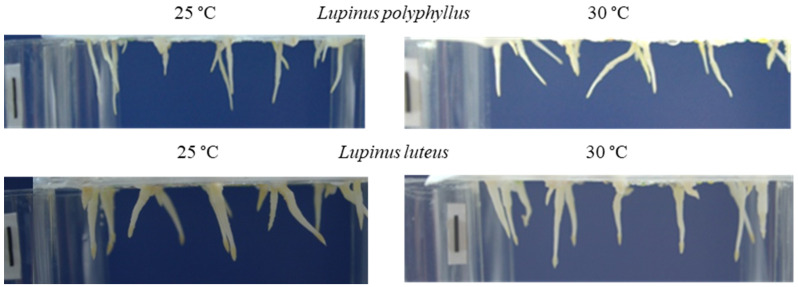
Spatial orientation of the initial roots of *L. polyphyllus* and *L. luteus* seedlings at 25 °C and 30 °C after 48 h. Scale bar, 10 mm.

After 48 h of seedling growth, the spatial orientation and growth direction of the roots of both lupines depended on the temperature: the angle of curvature of the primary roots of the invasive *L. polyphyllus* with respect to the gravitational vector was 6.2° at 25 °C, and 20.8° at 30 °C. The initial roots of the non-invasive *L. luteus* showed a better orientation towards the gravity vector at 30 °C (Table 1, Figure 1).

#### 2.1.2. Gravitropic Response of Initial Roots to 90° Reorientation

**Figure 2:**
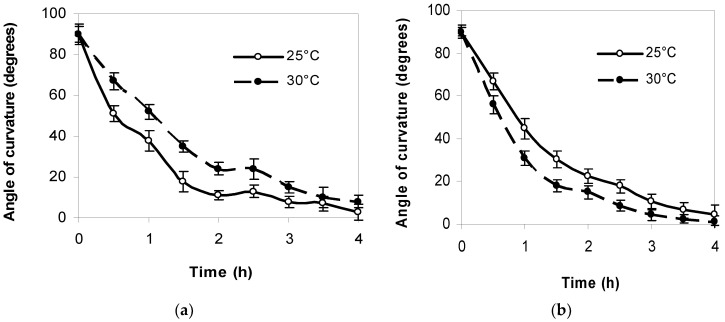
The dynamics of gravitropic response of *L. polyphyllus* (**a**) and *L. luteus* (**b**) roots to 90° reorientation at 25 °C and 30 °C.

The strongest root response to gravitropic irritation in both lupine species was found to occur within the first hour. The gravitropic bending of *L. polyphyllus* roots after 1 h was 16° greater at 25 °C than at 30 °C. The gravitropic bending of *L. luteus* roots was more intensive at 30 °C. The gravitropic response of the roots of both lupine species to a 90° reorientation was closer to the direction of gravity after 4 h (Figure 2).

#### 2.1.3. Growth of Primary Roots of 7-Day-Old Seedlings at 25 °C and 30 °C

**Figure 3:**
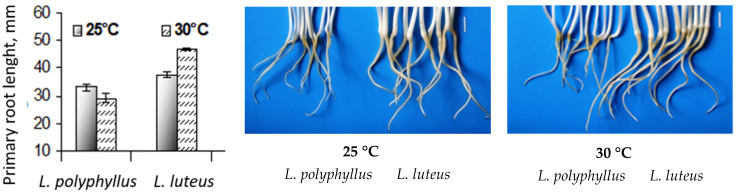
Effect of 25 °C and 30 °C temperature on root growth parameters of seven-day-old seedlings of two lupine species. Scale bar, 10 mm.

Morphometric studies showed that the length of roots of the invasive lupine grown at 30 °C for seven days was approximately 12% lower than that of the plants grown at 25 °C (Figure 3), while the roots of the non-invasive lupine grew up to 13% longer at 30 °C.

We found that the root-to-shoot ratio of both species decreased at 30 °C (Table 2). This index, in the case of *L. polyphyllus,* decreased crucially by 65% and in the case of *L. luteus* by 22%.

#### 2.1.4. Root Apex Development at 25 °C and 30 °C

**Figure 4:**
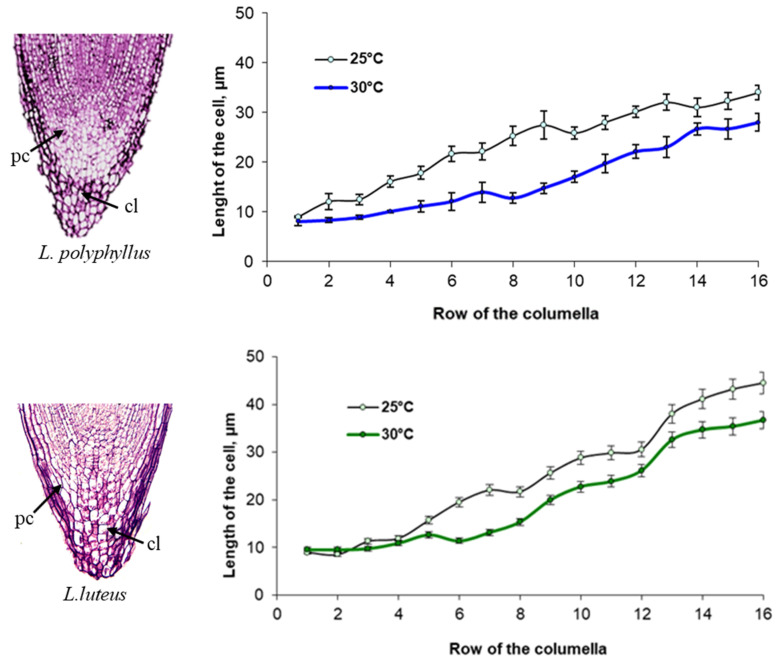
Impact of 25 °C and 30 °C temperature on the length of cells in the columella (cl) rows of the primary root cap (pc) (from the initial cells) of *L. polyphyllus* and *L. luteus*.

Cytomorphological analysis of the root cap columella of *L. polyphyllus* showed that the length of the cells in the individual rows of the columella varied with temperature (Figure 4). From the seventh row of the columella onwards, cell length increased more at 25 °C than at 30 °C. The changes in cell length in the *L. luteus* columella were substantially different from that of *L. polyphyllus.* The cell length of the columella at 30 °C was greater starting from the second row onwards. This trend was observed in all the following rows.

**Figure 5:**
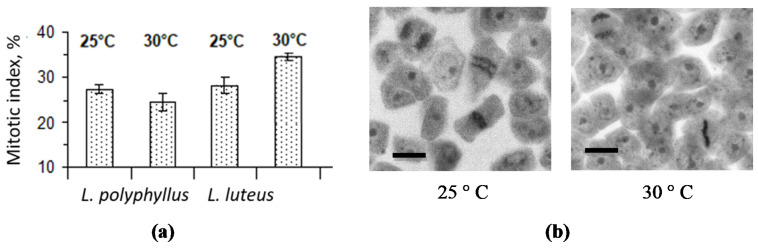
Effect of 25 °C and 30 °C temperatures on *L. polyphyllus* and *L. luteus* root apex meristem cells mitotic activity (**a**), fragments of primary root apical meristem pressed preparations from *L. polyphyllus* seedlings (**b**). Scale bar, 20 μm.

Determination of the cell division mitotic index (MI) in *L. polyphyllus* root apical meristem preparations showed that the cell MI value decreased by 12% in the test variant at 30 °C as compared with 25 °C (Figure 5a). Contrary, the calculation of MI in the non-invasive *L. luteus* root apical meristem indicated a significant increase at 30 °C. By observing the cross-sections of the invasive lupine root apex, we determined that meristem cells occurred in the prophase, metaphase and some even in the anaphase in the test variant at 25 °C, whereas most cells of plants grown at 30 °C were found in the prophase (Figure 5b).

### 2.2. The Development of 30-Day-Old Lupine Roots at 25 °C and 30 °C

**Figure 6:**
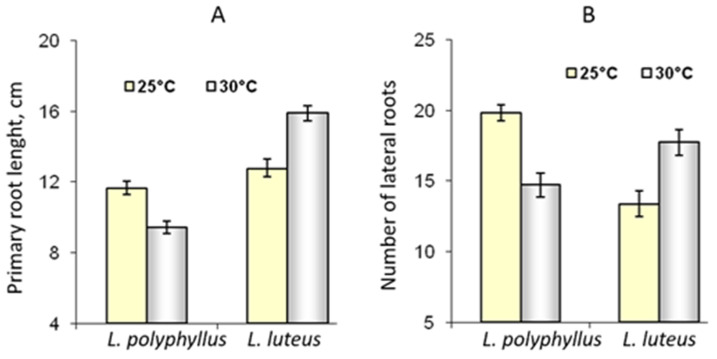
Effect of 25 °C and 30 °C temperature on the length of primary roots (**A**) and the number of lateral roots (**B**) of invasive *L. polyphyllus* and non-invasive *L. luteus* plants grown in soil for 30 days.

**Figure 7:**
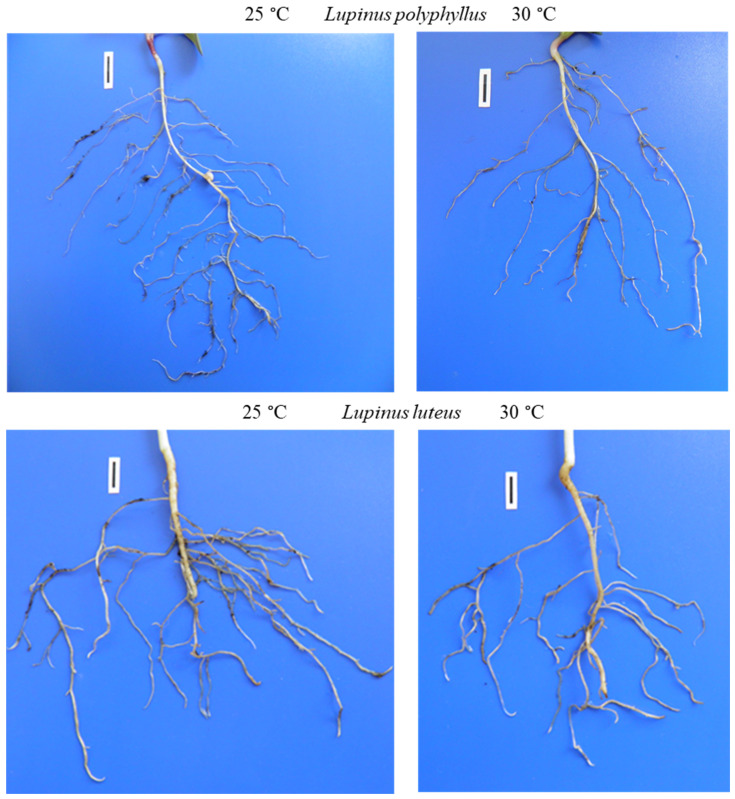
Roots of invasive *L. polyphyllus* and non-invasive *L. luteus* grown in the soil for 30 days. Scale bar, 10 mm.

The data of the morphometric measurements showed that simulated 5 °C warming affected invasive *Lupinus polyphyllus* root formation:primary root length decreased by 14% and the number of lateral roots by 16%. The length of the primary root and the number of lateral roots of non-invasive *L. luteus* were higher at 30 °C (Figure 6 and Figure 7).

## 3. Discussion

Temperature is one of the most important variables affecting plant growth. The effect of elevated temperature on aboveground plant parts has been well studied, while the effect on roots is less understood [2,17]. Roots need an optimal temperature range to grow and function properly. In general, the optimum root temperature is usually lower than the optimum shoot temperature. Literature shows that the effect of increasing temperature on root growth of plant seedlings can be either stimulatory, inhibitory or, once the optimum temperature is reached, initially stimulatory and then inhibitory [9,38,39]. In particular, it is important to study plants with different RSAs to understand the response of root development to temperature changes [1,2]. During seed germination, the growth of the seedling’s primary root and its ability to orient itself in space (gravitropism) are critical characteristics for seedling establishment and survival [35]. To answer the question of whether there are differences in the ability of the primary root of the two lupine species to respond to gravity and whether elevated temperature influences this process, an analysis of the direction of root growth in relation to gravity was conducted. This study showed that after 48 h of growth, the spatial orientation and subsequent growth direction of both lupine roots depended on the ambient temperature:angle of curvature of the initial roots of the invasive *L. polyphyllus,* with respect to the gravitational vector, was 6.2° at 25 °C, and 20.8° at 30 °C. Thus, the initial roots of the non-invasive *L. luteus* were better oriented towards the Earth’s gravity vector at 30 °C.

The vertical orientation of emerging roots is typically the first response of plants to gravity [40,41]. Sensing of the gravity stimulus ultimately triggers a signaling network orchestrated by the phytohormone auxin, which is key to the coordination of directional root growth in response to gravity [42,43,44]. Although root gravitropism has been studied extensively, no conclusive data on the onset of gravisensing is established. The inception of gravisensitivity in flowering plant roots after various periods of static orientation (gravistimulation) of imbibed seeds was studied [7,37,43]. Their results indicate that after gravistimulation (90° reorientation), gravitropic bending of flowering plant roots was established in 6 h along the gravity vector. These results well coincide with ours. Our data showed that after 1 h of gravistimulation (90° reorientation), the gravitropic bending of *L. polyphyllus* roots was 16° greater at 25 °C than at 30 °C; differently, the *L. luteus* roots response was more intensive at 30 °C. The gravitropic response of the initial roots of both lupine species to a 90° reorientation was closer to the direction of gravity after 4 h, both at 25 °C and at 30 °C. These data suggest that the initial roots of invasive lupines are less able to grow in the gravitational direction in a 5 °C warmer environment. Thus, dependence between the increase of environmental temperature and the inception of root gravitropic competence was determined. However, these parameters are not applicable to the description of RSA with complex geometry.

It has been shown that the effect of elevated temperature on the root growth of plant seedlings can be either activating or inhibiting in plants with a higher proportion of roots [2]. The morphometric tests carried out in this study showed that after seven days, the primary root growth of invasive lupines slowed down by 12% at 30 °C as compared to plants grown at 25 °C, while the root growth of non-invasive lupines accelerated by 13% at 30 °C. Elevated temperature is associated with a reduced root-to-shoot ratio, and a further increase in temperature limits root development and alters RSA [10]. We determined that the root-to-shoot ratio was reduced in both species at 30 °C; however, the roots of *L. luteus* were less sensitive to warming temperatures. It was obvious that this index, in the case of *L. polyphyllus,* decreased crucially.

Literature data indicate that the size of the root cap, the proportion of the columella in a root cap and meristem cell division were related to the growth of the roots [44,45]. It is known that the apical root growth correlated with the size of the columella and the number of cap cells in the plant root apex [46]. An increase in temperature promotes the initial growth of the roots of *Arabidopsis* seedlings and, at the same time, affects the elongation of columella cells [40]. In the current study, cytomorphological analysis of the root cap columella of *L. polyphyllus* showed that the length of the cells in the individual rows of the columella varied with temperature (Figure 4)—the increase of columella cell length was more intensive at 25 °C than at 30 °C. Nevertheless, *L. polyphyllus* cell length was greater at 30 °C already from the second row of the columella onwards. The apical meristem of roots provides cell regeneration, and the transition zone between the meristem and the cell extension zone enables the apex, directly or indirectly through the secondary signal, to sense changing environmental parameters and respond to changes in cell division [47,48]. Furthermore, anatomical-cytological analysis of apical meristem cells in the invasive lupine root apex showed that cell division was intense at a lower temperature. By observing the cross-sections of root apex, we determined that in the test variant at 25 °C, meristem cells occurred in the prophase, metaphase and some even in the anaphase, whereas most cells of plants grown at 30 °C were found in the prophase.

The data on differences in root size of *L. polyphyllus* and *L. luteus* resulting from temperature change suggests that the elevated temperature may be more difficult for invasive lupines to adapt to. The architecture of the root system is determined by the development of both primary and lateral roots [49,50]. The plant root system takes up water and dissolved nutrients from the soil; therefore, the size and extent of the root system have important implications for plant development [7,51]. Our results show that the two species of lupine seedlings grown in the soil for 30 days responded differently to changes in temperature. The most significant changes were observed in root length and lateral root formation. Plants of the invasive lupine had a larger root system at 25 °C, and the root size of non-invasive lupine generally increased at 30 °C. Under the elevated temperature, non-invasive plants produced more extensive root systems.

Our results provide key information concerning the elevated temperature on the formation of root architecture of two lupine species and suggest that the elevated temperature affects species invasiveness. In the early stages of growth (after 48 h), the spatial orientation of the initial roots of both lupines depended on the temperature—the angle of curvature of the initial roots of *L. polyphyllus* was closer to the gravity vector than *L. luteus* at 25 °C. The initial roots of the non-invasive *L. luteus* showed a better orientation towards the gravity vector at 30 °C. These processes were important for the subsequent formation of root architecture—the dynamics of gravitropic response of *L. polyphyllus* and *L. luteus* initial roots to 90° reorientation showed that the gravitropic bending of *L. luteus* roots was more intensive at 30 °C. Simulated warming (5 °C) affected *L. polyphyllus* root formation as the initial roots were characterized by disrupted gravitropic orientation to the gravity vector; the cell division mitotic index (MI) of root apical meristem decreased by 12% at 30 °C as compared with 25 °C. The temperature of 30 °C triggered the non-invasive *L. luteus* root development, formation and spatial orientation, both in the initial and later stages of development. After 30 days of growth, seedlings of the two lupine species responded differently to elevated temperature—the invasive lupine formed a larger root system at 25 °C, and the non-invasive lupine root size increased at 30 °C. Bearing in mind that global warming tends to enhance species invasiveness and the northward spread, among other issues, these findings provide important information on the effect of increased temperature on the formation of plant root architecture and suggest that elevated temperature alters the invasiveness of alien species due to changes in root architecture.

## 4. Materials and Methods

### 4.1. The Initial Root Growth at 25 °C and 30 °C

Two different lupine species—invasive *L. polyphyllus* and non-invasive *L. luteus* [52]—seeds were harvested in a natural environment in Lithuania and used as plant material. Seeds were soaked for 5 h in tap water at room temperature and then germinated in climate chambers (Climacell, Czech Republic) at 90% relative humidity in the dark at two different temperatures: at 25 °C (optimal temperature for lupine) and 30 °C (simulated climate warming temperature) [53]. For root system architecture exploration, seeds were sown in 7 cm diameter pots containing a mixture of vegetable compost 90%, peat 9%, ash of deciduous trees 1% and fertilizer NPK and grown 30 days in growth chambers with 12 h light/dark photoperiod, at 25 °C and 30 °C.

### 4.2. The Measurement of the Angle of Root Curvature

The assessment of root-growth patterns is based on the measurement of angular deviation of the root tip from the vertical axis. For assay of roots gravitropic response, 30 soaked seeds of both lupines were planted in gaps in transparent plexiglass boxes filled with distilled water so that protruding roots could grow freely downwards, i.e., towards the action of the gravitational force. The seedlings grew in the germinators at 25° and 30 °C, and relative humidity of 90% in the dark. The angle of root curvature towards the vector of gravity was measured at the 48th hour of growth.

### 4.3. Determination of Gravitropic Response of Roots to 90° Reorientation

Seeds were germinated on wet filter paper for 21 h and then planted on a sterile control medium (1% agar [*w*/*v*]) in square Petri dishes. The seeds with initial roots were fastened by agar and oriented so that roots could orient freely along the agar surface for 24 h in a vertical orientation. The dynamics of root curvature as an angle towards the gravitropic vector were measured at the 4-h period of the reorientation in a 90-degree position.

### 4.4. Morphometrical Tests

Measurements of root length and root-to-shoot ratio were performed on 7-day-old seedlings grown in tap water in the dark at 25 °C and 30 °C. The length of the primary root and the number of lateral roots of the two species of lupine were measured after 30 days of growth at 25 °C and 30 °C in soil.

### 4.5. Anatomical-Cytometrical Analysis of Primary Root Development

#### 4.5.1. Cytometrical Investigations

Primary roots were excised from roots of 10 seedlings (7-day-old). The prepared samples were fixed in a formalin:acetic acid:ethanol (1:1:20) (FAA) mixture, dehydrated in a graded ethanol series, embedded in paraffin and cut with a rotary microtome Leica RM2125 into 10–15 µm sections. Serial longitudinal sections were stained with periodic acid-Schiff’s reagent, and the length of statocytes in the columella rows of the root cap were measured with a light microscope and a digital video camera (Olympus) (DP-11). The images were analyzed using the SigmaScan Pro (Jandel Scientific Software) program.

#### 4.5.2. Determination of Mitotic Index

For estimation of primary root apical meristem cells’ mitotic activity, the roots were fixed in acetic acid:ethanol mixture (1:3). After 4 days of fixation, roots were washed from the mixture; the apical meristem zone was excised and dyed with acetocarmine, whereas cell walls were macerated with chloral hydrate [54]. In temporary squash preparations by a light microscope (Nikon Eclipse 80i), 6 cell mitoses phases were counted and mitotic index (MI) calculated. MI—cell number in mitosis per 1000 cells of the analyzed object (expressed in per mille ^o^/_oo_). MI = (M/N) 1000, where M—number of mitoses, N—cell number. For each variant, 20 primary root apical meristems were analyzed.

### 4.6. Statistical Analysis

Tests were provided with three biological replicates. For morphometrical measurements, roots of 40 seedlings were analyzed for each variant. The data presented are mean values ± standard deviation of three experiments with four replicates in each. The data were statistically examined using analysis of variance (ANOVA) and tested for significant mean differences (*p* < 0.05) using Tukey’s test.

## 5. Conclusions

Elevated temperature impacted the formation of root architecture of two lupine species while influencing their invasiveness.

During the early stages of growth, the spatial orientation of the initial roots was temperature-dependent: at 25 °C, the angle of curvature of the initial roots of *L. polyphyllus* was closer to the gravity vector than that at 30 °C, while *L. luteus* were better oriented towards the gravity vector at 30 °C.

The dynamics of the gravitropic response of initial roots to 90° reorientation confirmed that the gravitropic bending of *L. luteus* roots was more intense at 30 °C; meanwhile, *L. polyphyllus* was at 25 °C.

The simulated warming (5 °C) had an effect on *L. polyphyllus* root formation: the mitotic index of cell division in the root apical meristem was reduced by 12% at 30 °C compared to 25 °C.

After 30 days of cultivation at different temperatures, the root system of the invasive lupine was better developed at 25 °C, whereas the root size of the non-invasive lupine increased at 30 °C.

The current study provides important information on the effect of elevated temperature on the formation of plant root architecture and suggests that global warming is altering the invasiveness of alien species through changes in root architecture.

## Figures and Tables

**Table 1:** Influence of 25 °C and 30 °C temperatures on the angle of curvature of the initial roots of *L. polyphyllus* and *L. luteus* seedlings grown vertically for 48 h.

Plant Species	*Lupinus polyphyllus*		*Lupinus luteus*	
Temperature	25 °C	30 °C	25 °C	30 °C
Angle of curvature, degrees	6.2 ± 0.53 a	20.8 ± 0.95 b	14.2 ± 1.21 c	6.8 ± 0.43 a

Values presented are the mean values of four replications with standard deviation. Different lowercase letters indicate significant differences between test variants at *p* < 0.05.

**Table 2:** Influence of 25 °C and 30 °C temperature on root-to-shoot ratio of the seven-day-old seedlings of *L. polyphyllus* and *L. luteus*.

Plant Species	*Lupinus polyphyllus*		*Lupinus luteus*	
Temperature	25 °C	30 °C	25 °C	30 °C
Root-to-shoot ratio	0.182 ± 0.03 a	0.063 ± 0.01 b	0.217 ± 0.03 a	0.169 ± 0.01 a

Values presented are the mean values of four replications with standard deviation. Different lowercase letters indicate significant differences between test variants, at *p* < 0.05.

## Data Availability

The data supporting reported results can be found in scientific reports of the Laboratory of Plant Physiology of Institute of Botany of Nature Research Centre, where archived datasets generated during the study are included.
